# An Anti‐CD147 Antibody−Drug Conjugate Mehozumab‐DM1 is Efficacious Against Hepatocellular Carcinoma in Cynomolgus Monkey

**DOI:** 10.1002/advs.202410438

**Published:** 2025-02-22

**Authors:** Wan Huang, Liping Zhong, Ying Shi, Qingzhi Ma, Xiangmin Yang, Hongmei Zhang, Jing Zhang, Ling Wang, Kun Wang, Jingzhuo Li, Jie Zou, Xu Yang, Liu Yang, Qingmei Zeng, Lin Jing, Zhi‐Nan Chen, Yongxiang Zhao

**Affiliations:** ^1^ Department of Cell Biology National Translational Science Center for Molecular Medicine Fourth Military Medical University Xi'an Shaanxi 710032 China; ^2^ State Key Laboratory of New Targets Discovery and Drug Development for Major Diseases Xi'an Shaanxi 710032 China; ^3^ State Key Laboratory of Targeting Oncology National Center for International Research of Biotargeting Theranostics Guangxi Key Laboratory of Biotargeting Theranostics Collaborative Innovation Center for Targeting Tumor Diagnosis and Therapy Guangxi Medical University Nanning Guangxi 530021 China; ^4^ Department of Clinical Oncology Xijing Hospital Fourth Military Medical University Xi'an Shaanxi 710032 China; ^5^ Department of Pathology Xijing Hospital The Fourth Military Medical University Xi'an Shaanxi 710032 China; ^6^ Department of Health Statistics School of Preventive Medicine Fourth Military Medical University Xi'an Shaanxi 710032 China

**Keywords:** antibody‐drug conjugate, cell necroptosis, hepatocellular carcinoma, therapeutic resistance

## Abstract

Effective treatment strategies are urgently needed for hepatocellular carcinoma (HCC) patients due to frequent therapeutic resistance and recurrence. Antibody‐drug conjugate (ADC) is a specific antibody‐drug conjugated with small molecular compounds, which has potent killing activity against cancer cells. However, few ADC candidates for HCC are undergoing clinical evaluation. CD147 is a tumor‐associated antigen that is highly expressed on the surface of tumor cells. Here CD147 is found significantly upregulated in tumor tissues of HCC. Mehozumab‐DM1, a humanized anti‐CD147 monoclonal antibody conjugated with Mertansine (DM1) is developed. Mehozumab‐DM1 is effectively internalized by cancer cells and demonstrated potent antitumor efficacy in HCC cells. In vivo evaluation of Mehozumab‐DM1 is conducted in a CRISPR‐mediated *PTEN* and *TP53* mutation cynomolgus monkey liver cancer model, which is poorly responsive to sorafenib treatment. Mehozumab‐DM1 demonstrated potent tumor inhibitory efficacy at doses of 0.2 and 1.0 mg kg^−1^ treatment groups in cynomolgus monkey. No treatment‐related adverse reactions or body weight loss are observed. Interestingly, Mehozumab‐DM1 treatment induced RIPK‐dependent tumor cell necroptosis through inhibiting IκB kinase/NF‐κB pathway. In conclusion, Mehozumab‐DM1 potently inhibits hepatoma through effective internalization to release payload and inducing cell necroptosis to enhance the bystander effect, which is a promising treatment for refractory HCC.

## Graphical abstract

Antibody‐drug conjugates are among the fastest‐growing drugs in oncology. It combines the potent killing activity of small molecular drugs and the high specific targeting of antibody drugs. Our in vitro and in vivo studies suggest that anti‐CD147 ADC Mehozumab‐DM1 potently inhibits hepatocellular carcinoma, and combining CD147‐ADCs and targeted therapy may provide optimal therapeutics for patients with refractory liver cancer.

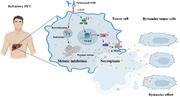

## 1 Introduction

Liver cancer is the second leading cause of cancer‐related deaths in China yet without effective treatment. Despite significant therapeutic progress, including immune checkpoint inhibitors and targeted therapy, such as sorafenib, lenvatinib, atezolizumab plus bevacizumab, tremelimumab plus durvalumab, and others,^[^ ^1^, ^2^ ^]^ the majority of patients with HCC still suffer from therapeutic resistance, side effects, and recurrence.^[^ ^3^, ^4^ ^]^ Antibody‐drug conjugates (ADCs) use a chemical linker to conjugate cytotoxic drugs to a specific monoclonal antibody (mAb), thereby combining the potent killing activity of a small molecule drug and the highly specific targeting of an antibody drug.^[^ ^5^ ^]^ Unlike traditional chemotherapeutic agents, ADCs are designed to deliver potent cytotoxic substances into tumors with high specificity, leading to remarkably reduced side effects.^[^ ^6^, ^7^, ^8^ ^]^

Mehozumab is a humanized anti‐human CD147 antibody. CD147 is an important adhesion molecule that plays a significant role in numerous pathological processes.^[^ ^9^ ^]^ Many studies have reported CD147 overexpression in various carcinomas, with a significantly higher positivity rate and stronger expression intensity than that in normal tissues.^[^ ^10^ ^]^ Our previous studies showed that Licartin ([^131^I] monoclonal antibody for CD147) was a safe and effective drug in treating patients with HCC and preventing post‐orthotopic liver transplantation tumor recurrence.^[^ ^11^, ^12^ ^]^ However, radiological protection is required during the Licartin treatment. Therefore, ADC treatment for HCC would be more suitable for widespread clinical use.

Non‐human primates (NHPs) are ideal animal models for drug development. However, the primary challenge remains that NHPs have yet to be used as in vivo pharmacodynamic models to simulate human diseases, such as cancer.^[^ ^13^ ^]^ Our previous study developed an in situ gene‐editing approach to induce efficient loss‐of‐function mutations in *PTEN* and *TP53* for the rapid modeling of primary and metastatic liver tumors using CRISPR/Cas9 in adult cynomolgus monkeys. The best mutation efficiencies for *PTEN* and *TP53* were 74.71% and 74.68%, respectively.^[^ ^14^ ^]^ Thus, this cynomolgus monkey model holds promise as a powerful yet feasible tool to construct human liver cancer models in adult NHPs.

Systemic therapy is the mainstay of refractory HCC treatment, and ADC is a promising treatment strategy.^[^ ^4^ ^]^ In this study, we developed an anti‐CD147 ADC, Mehozumab‐DM1, comprising a humanized CD147 antibody as the targeting component with the potent cytotoxic drug DM1 as the payload component, conjugated via SMCC, a non‐cleavable thioether linker. Here, we demonstrate that the Mehozumab‐DM1 is a potential therapeutic drug against liver cancer.

## 2 Results

### 2.1 Expression of CD147 was Significantly Upregulated in Tumor Tissues of HCC and Correlated with Targeted Therapy Resistance

**Figure 1:**
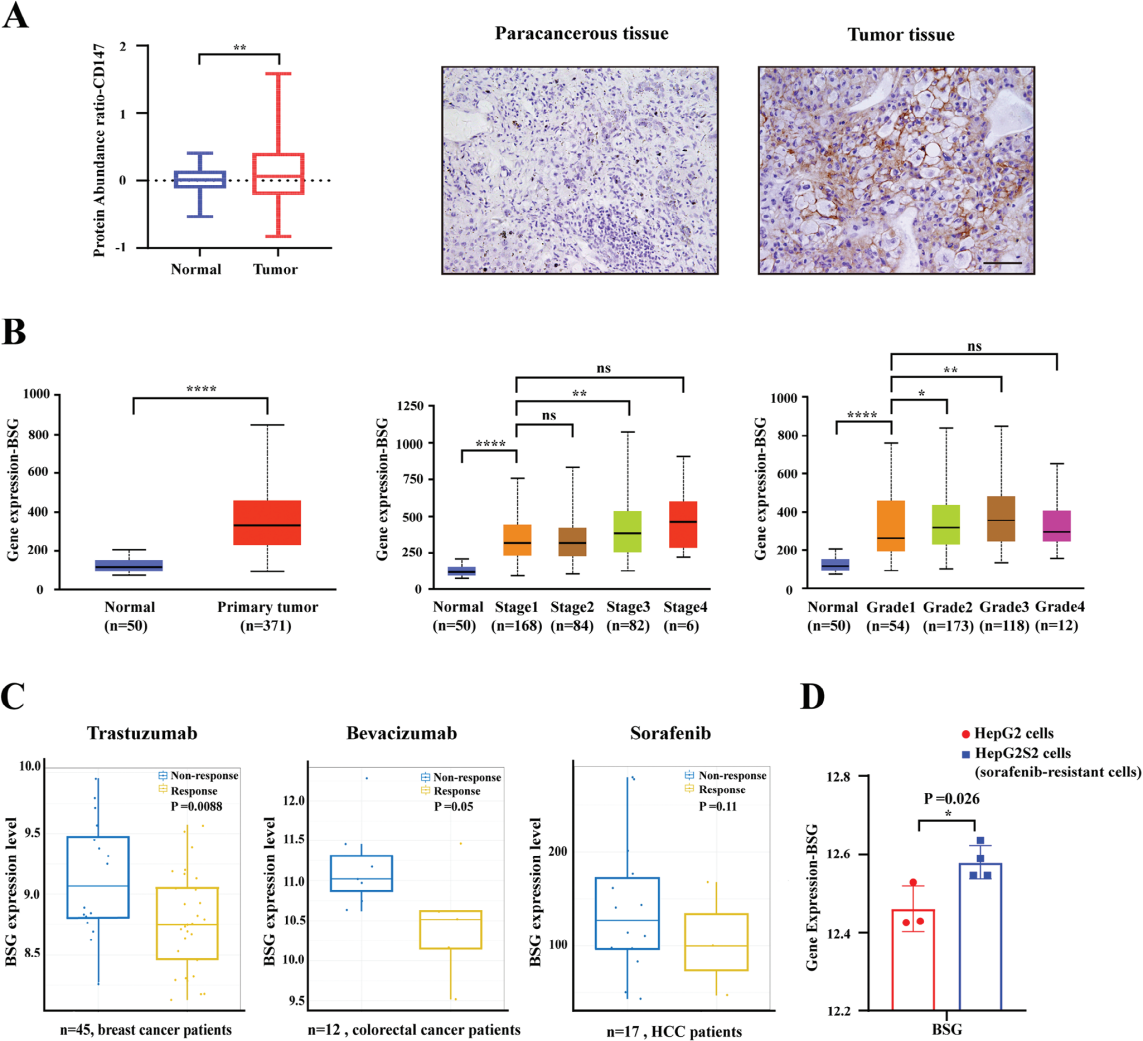
Expression of CD147 was significantly upregulated in tumor tissues of HCC and correlated with targeted therapy resistance. A) CD147 protein levels were quantitatively compared in tumor and normal tissues of HCC patients based on the CPTAC dataset(*n* = 153). Representative image of IHC of CD147 in para‐tumor and tumor tissues from patients with HCC. Scale bars represent 50 µm. B) Transcription level of CD147 (BSG) in the normal tissue, tumor tissue, different stages of HCC according to AJCC staging, and different pathological grades of HCC. Data from the TCGA dataset. C) Gene expression of CD147 (BSG) was quantitatively compared in the tumor tissues from trastuzumab (*n* = 45), bevacizumab (*n* = 12) and sorafenib (*n* = 17) non‐response patients and response patients based on CTR‐DB dataset. D) BSG was quantitatively compared in sorafenib‐resistant HepG2 cells and HepG2 cells based on the GEO dataset. Independent Student's *t*‐test was used to compare the mean expression level of two different groups. ^*^p < 0.05, ^**^p < 0.01, ^***^p < 0.001. NS, is not significant.

CD147 has been proven to be an important marker in the progression of HCC.^[^ ^15^ ^]^ To fully elaborate on the expression of CD147 in HCC patients, we analyzed the protein expression of CD147 based on the CPTAC dataset (https://proteomics.cancer.gov/programs/cptac). We found significantly higher levels of CD147 in the tumor tissues than in the normal tissues of patients with HCC (*p* < 0.01), and immunohistochemical staining demonstrated specifically high expression of CD147 on the surface of hepatoma cells (**Figure** **1**A). Data from the TCGA dataset (https://www.cancer.gov/ccg/research/genome‐sequencing/tcga) indicated significant upregulation of CD147 transcription in the tumor tissues of HCC (*p* < 0.0001). Moreover, CD147 was remarkably elevated in the early stage of HCC according to the American Joint Committee on Cancer staging criteria (*p* < 0.0001) and tumor pathology grading (*p* < 0.0001) (Figure 1B).

Then the correlation of CD147 and refractory HCC was analyzed. The analysis from the TISCH2 scRNA‐seq database (http://tisch.comp‐genomics.org/) demonstrated significantly higher levels of CD147 expression in patients who received PDL‐1/CTLA4 treatment compared with patients who did not receive immunotherapy (*p* < 0.0001; Figure S1A, Supporting Information). Moreover, higher expression of CD147 was found in trastuzumab, bevacizumab, and sorafenib non‐response patients than that in response patients (data from the CTR‐DB dataset,^[^ ^16^ ^]^ *P* = 0.0088, 0.05, and 0.11, respectively), and CD147 expression was significantly higher in sorafenib‐resistant HepG2 cells (data from the GEO dataset, *P* = 0.026) (Figure 1,D). Survival analysis based on the Kaplan–Meier Plotter dataset (https://kmplot.com/analysis/) revealed that higher expression of CD147 correlated with poor overall survival (*P* = 0.0013), poor progression‐free survival (*P* = 0.014), and poor recurrence‐free survival (*P* = 0.0059) (Figure S1B, Supporting Information).

### 2.2 Mehozumab‐DM1 is Effectively Internalized and Exhibits Antitumor Efficacy in HCC Cells

**Figure 2:**
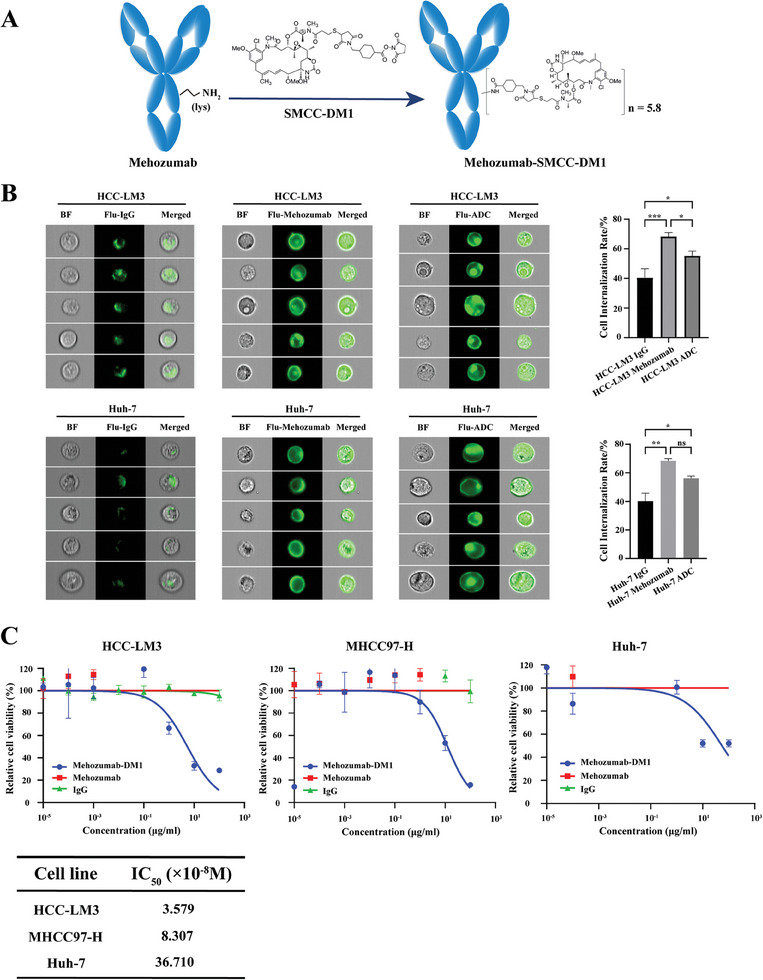
Mehozumab‐DM1 is effectively internalized and exhibits antitumor efficacy in HCC cells. A) Diagram of the preparation process of Mehozumab‐DM1. Mehozumab was conjugated to DM1 via the non‐cleavable linker SMCC, forming Mehozumab‐DM1, with an average drug‐to‐antibody ratio (DAR) of 5.80. B) Representative imaging flow cytometry images showing cellular internalization of IgG, Mehozumab, and Mehozumab‐DM1 labeled with FITC (green) in HCC‐LM3 and Huh‐7 HCC cell lines (left). Signal intensity analysis for Mehozumab‐DM1 antibody‐mediated cell internalization (right). Independent Student's *t* test was used to compare the mean expression level of two different groups. ^*^p < 0.05, ^**^p < 0.01, ^***^p < 0.001. ns, not significant. BF, bright field; IgG, immunoglobulin G. C) Cell Counting Kit‐8 experiments were conducted to evaluate the inhibitory efficacy of Mehozumab‐DM1 on hepatocellular carcinoma cell lines HCC‐LM3, MHCC97‐H, and Huh‐7. Mehozumab‐DM1, Mehozumab, and IgG with different concentration gradients were added to the cells and treated for 72 h. IC_50_ values for three cell lines were calculated in the table.

Mehozumab was derived from a murine HAb18 antibody.^[^ ^17^, ^18^, ^19^ ^]^ A Humanized Mehozumab antibody was engineered by grafting complementarity‐determining regions into the corresponding regions of a fixed human framework scaffold in our laboratory (patent CN201910796766.8, https://pss‐system.cponline.cnipa.gov.cn/). Mehozumab was conjugated to DM1 via the non‐cleavable linker SMCC, forming Mehozumab‐DM1, with an average of 5.80 DM1 molecules conjugated to per antibody (**Figure** **2**A). HPLC analysis revealed that the ADC purity was > 99.99% (Figure S2A, Supporting Information). Drug‐to‐antibody ratio (DAR) of the ADC was detected by quadrupole Time‐of‐Flight LC/MS. The mass spectrogram for unconjugated Mehozumab and Mehozumab‐DM1 were shown in Figure S2B (Supporting Information), and the calculated DAR was 5.80. UV spectrophotometry analysis revealed an average DAR of 5.64, and a representative absorbance spectrum is shown in Figure S2C (Supporting Information). Moreover, the binding affinity of Mehozumab‐DM1 to CD147 was comparable to the parent antibody Mehozumab with a parallel SPR equilibrium dissociation constant of 3.595 and 3.411 nmol L^−1^, respectively (Figure S2D, Supporting Information). Immunohistochemical staining further demonstrated that the affinity and specificity of Mehozumab‐DM1 binding to CD147 in tumor tissues of our cynomolgus monkey model was comparable to the naked Mehozumab antibody (Figure S2E, Supporting Information). Therefore, Mehozumab‐DM1 retained a high affinity to CD147 and was not significantly affected by conjugation to DM1.

To determine whether Mehozumab‐DM1 underwent rapid transit into cell endosomes for payload release, we quantified the cellular internalization rate of Mehozumab‐DM1 in HCC cell lines using imaging flow cytometry. Confocal fluorescent images revealed that both Mehozumab and Mehozumab‐DM1 were rapidly internalized by LM3 and Huh‐7 HCC cells through receptor‐mediated endocytosis after a 1 h incubation (Figure 2B). Quantitative analysis showed that the cellular internalization rates of Mehozumab and Mehozumab‐DM1 were significantly higher than that of immunoglobulin G (IgG) in the HCC cell lines (Figure 2B). We performed cell proliferation assays to evaluate the effect of Mehozumab‐DM1 on hepatocellular carcinoma cell lines HCC‐LM3, MHCC97‐H, and Huh‐7, which were found to highly express CD147 according to our previous studies. The result of Cell Counting Kit‐8 analysis showed that Mehozumab‐DM1 significantly suppressed the cell viability in all three cell lines and demonstrated potent cell‐killing activity, with IC_50_ values of 3.597, 8.307 and 36.71 × 10^−8^ m for HCC‐LM3, MHCC97‐H, and Huh‐7 cells, respectively (Figure 2C).

### 2.3 In Vivo Inhibitory Efficacy of Mehozumab‐DM1 in a CRISPR‐Mediated Cynomolgus Monkey Liver Cancer Model

**Figure 3:**
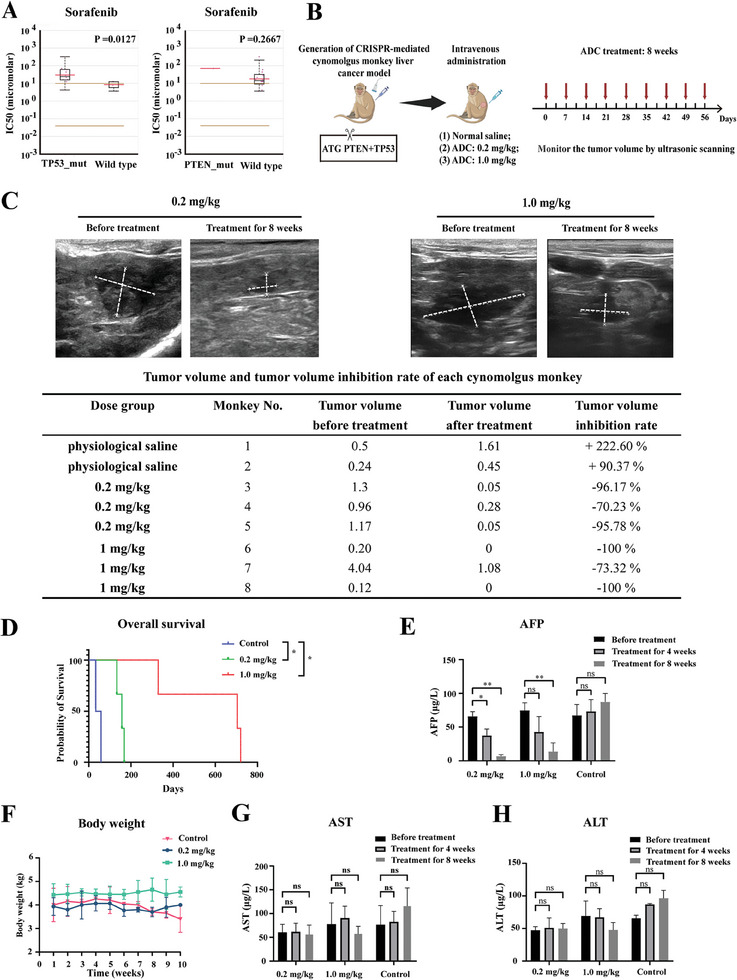
In vivo inhibitory efficacy of Mehozumab‐DM1 in CRISPR‐mediated cynomolgus monkey liver cancer model. A) Correlation of TP53 (left) and PTEN (right) mutation and IC_50_ of sorafenib in HCC cell lines (data from The Genomics of Drug Sensitivity in Cancer Project, TP53‐mut: *n* = 10, WT: *n* = 5. PTEN‐mut: *n* = 1 WT: *n* = 14). B) Schematic design of in vivo study in CRISPR‐mediated cynomolgus monkey liver cancer model. C) Typical photos of ultrasonic scanning before and after ADC treatment for 8 weeks in Mehozumab‐DM1 intravenous injection 0.2 and 1.0 mg kg^−1^ group. The detailed data of tumor volume before and after treatment of each cynomolgus monkey, and tumor volume inhibition rate was shown in the table below. D) Kaplan Meier curves and log‐rank analyses to investigate the overall survival in cynomolgus monkey treatment with physiological saline, 0.2 and 1 mg kg^−1^ ADC. E) The level of AFP in peripheral blood of the cynomolgus monkey before and after the ADC treatment for 4 and 8 weeks in control and Mehozumab‐DM1 treatment 0.2 and 1.0 mg kg^−1^ groups. F) The body weight of the cynomolgus monkey was recorded for 10 weeks during ADC treatment. G,H) The level of AST and ALT in peripheral blood of the cynomolgus monkey before and after the ADC treatment for 4 and 8 weeks in control and Mehozumab‐DM1treatment groups. Independent Student's *t* test was used to compare the mean expression level of two different groups. ^*^p < 0.05, ^**^p < 0.01, ns, not significant. AFP: alpha‐fetoprotein; AST: Aspartate aminotransferase; ALT: Alanine aminotransferase.

We previously generated a CRISPR‐mediated *PTEN* and *TP53* mutation cynomolgus monkey liver cancer model.^[^ ^14^ ^]^ The *TP53* mutation was significantly related to a poor response to sorafenib in HCC cell lines (**Figure** **3**A, data from the Genomics of Drug Sensitivity in Cancer Project, https://www.cancerrxgene.org), and the *PTEN* mutation was reported as remarkably correlated with immunotherapy resistance.^[^ ^20^, ^21^, ^22^, ^23^ ^]^ Figure 3B presents a schematic of our in vivo study design. Briefly, the loss‐of‐function mutations in *PTEN* and *TP53* expressed by CRISPR/Cas9 adenoviruses were directly delivered to the livers of cynomolgus monkeys through the intrahepatic portal vein under ultrasound guidance to achieve in situ gene editing. When the tumor diameter reached 1.00 cm, qualified cynomolgus monkey liver cancer models were selected and randomly divided into an ADC intravenous injection 0.2 mg kg^−1^ group (*n* = 3), an ADC intravenous injection 1.0 mg kg^−1^ group (*n* = 3), and a physiological saline control group (*n* = 2). The ADC was administered once a week for 8 weeks. Tumor volumes were observed by ultrasound scans, and Figure 3C shows representative photos of ultrasound scans before and after the 8‐week ADC treatment. Table S1 (Supporting Information) shows detailed data on the tumor size for each cynomolgus monkey. We observed a marked shrinkage in tumor volume, with an overall response rate of 100% in the 0.2 and 1.0 mg kg^−1^ ADC treatment groups. The tumor volume of each cynomolgus monkey before and after treatment, and the tumor volume inhibition rate is shown in Figure 3C. The average tumor volume inhibition rate was 87.39% and 91.11% in the 0.2 and 1.0 mg kg^−1^ groups, respectively. One‐way analysis of variance (ANOVA) revealed a significant difference in the tumor volume inhibition rate between the control and treatment groups (*P* = 0.003), and the post‐hoc test showed significant differences in the tumor volume inhibition rate between the control group and the 0.2 mg kg^−1^ treatment group (*P =* 0.002) and between the control group and the 1.0 mg kg^−1^ treatment group (*P =* 0.002). Overall survival analysis using the log‐rank test revealed a correlation between ADC treatment at doses of both 0.2 and 1.0 mg kg^−1^ and prolonged survival of the model monkeys (*P* = 0.0389 and *P* = 0.0389, respectively; Figure 3D). Furthermore, the level of serum alpha‐fetoprotein (AFP) decreased markedly in the 0.2 and 1.0 mg kg^−1^ groups after 8 weeks of ADC treatment (*P* = 0.000155 and *P* = 0.003369, respectively; Figure 3E). Regarding safety, there was no treatment‐related body weight loss in either treatment group (Figure 3F) and no significant change in aspartate aminotransferase (AST) or alanine aminotransferase (ALT) levels after ACD treatment (Figure 3G,H), suggesting the absence of ADC treatment‐induced liver injury. Furthermore, no significant change in the red blood cell count, white blood cell count, platelet count, and percentage of eosinophils and lymphocytes were observed after ADC treatment (Table S2, Supporting Information), suggesting that this drug did not cause anemia or infection.

### 2.4 Cell Necroptosis Induced by Mehozumab‐DM1 in Tumor Tissues from Cynomolgus Monkey

**Figure 4:**
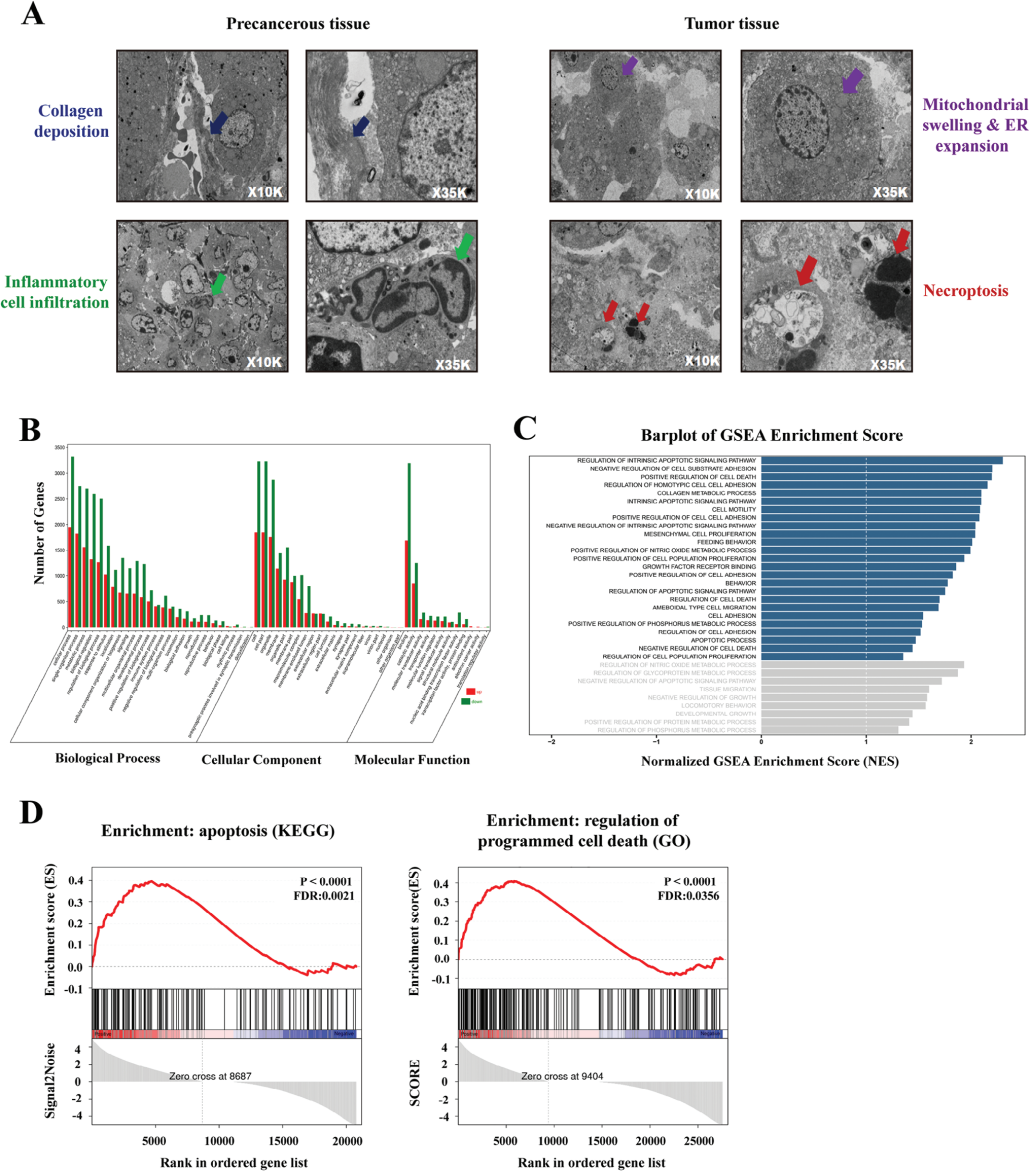
Cell necroptosis induced by Mehozumab‐DM1 in tumor tissues from cynomolgus monkey. A) Pathological changes in tumor tissues and precancerous tissues of cynomolgus monkeys after ADC treatment were detected by electron microscopy. ADC treatment led to obvious collagen deposition (blue arrow) and inflammatory cell infiltration (green arrow) in precancerous tissue (left). In tumor tissue (right), mitochondrial swelling and ER expansion in the cell (purple arrow), and typical cell necroptosis (red arrow) were observed. B) Total RNA from the tumor tissue of physiological saline control cynomolgus monkey and cynomolgus monkey treated with 1mg kg^−1^ ADC was extracted to carry out RNA‐seq. The functional interpretation of dysregulated RNA was annotated using GO analysis. C) Differential genes between control and ADC treatment cynomolgus monkey were enriched by Gene Set Enrichment Analysis (GSEA). Genes related to the intrinsic apoptotic and cell death signaling pathway rank high in their GSEA enrichment scores. D) GSEA to identify the apoptosis and programmed cell death signaling pathways enriched in differential genes between control and ADC treatment cynomolgus monkey.

Pathological changes in the tumor tissues of cynomolgus monkeys after ADC treatment were detected by H&E staining and electron microscopy. H&E staining showed distinct liquefactive necrosis in the tumor tissue of the 0.2 and 1.0 mg kg^−1^ ADC treatment groups (Figure S3, Supporting Information). Electron microscopy photos revealed obvious collagen deposition and inflammatory cell infiltration in precancerous tissue following the ADC treatment. In contrast, typical necroptosis was observed in tumor tissues after ADC treatment, and the tumor cells showed mitochondrial swelling and endoplasmic reticulum expansion (**Figure** **4**A).

To identify the mechanism by which Mehozumab‐DM1 suppresses liver cancer, we performed RNA sequencing (RNA‐seq) of total RNA extracted from the tumor tissue of cynomolgus monkey in the group treated with physiological saline and the group treated with 1.0 mg kg^−1^ ADC. The function of the dysregulated RNA was annotated using gene ontology (GO) analysis according to the domains of biological process, cellular component, and molecular function. The top GO term was a cellular process (in the biological process domain) (Figure 4B). Additionally, Gene Set Enrichment Analysis (GSEA) revealed that genes related to the intrinsic apoptotic and cell death signaling pathway were highly enriched (Figure 4C). Further analysis of signaling pathways by GSEA enrichment suggested that the molecular components of the cell apoptosis and programmed cell death signaling pathway were significantly correlated with differential genes between the control and ADC‐treated cynomolgus monkey (Figure 4D).

### 2.5 Mehozumab‐DM1 Treatment Induced RIPK‐Dependent Tumor Cell Necroptosis Through Inhibiting IκB Kinase/NF‐κB Pathway

**Figure 5:**
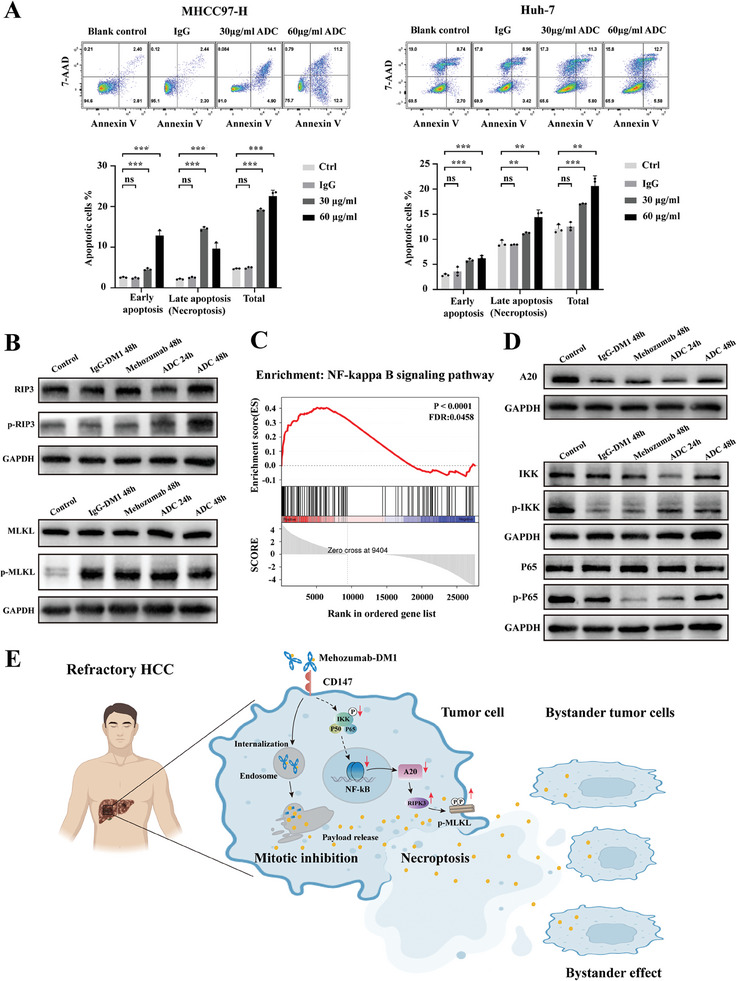
Mehozumab‐DM1 treatment induced RIPK‐dependent tumor cell necroptosis through inhibiting IκB kinase / NF‐κB pathway. A) Annexin V/7‐AAD apoptosis analysis by flow cytometry in MHCC97‐H cells (left) and Huh‐7 cells (right) after 30 and 60 µg mL^−1^ ADC treatment for 72 h. Independent Student's *t* test was used to compare the mean expression level of two different groups. ^*^p < 0.05, ^**^p < 0.01, ^***^ *p* < 0.001, ns, not significant. B) Immunoblotting analysis to detect the necroptosis markers RIPK3 and MLKL in Huh‐7 cells, after 24 and 48 h Mehozumab‐DM1 treatment. C) GSEA enrichment analysis of NF‐κB pathway in differential genes between control and ADC treatment cynomolgus monkey. D) Immunoblotting analysis of TNFAIP3/A20 (tumor necrosis factor alpha‐induced protein 3), phosphorylated IKK (inhibitor of kappa B kinase), and P65 (nuclear factor kappa B p65) after 24 and 48 h Mehozumab‐DM1 treatment. E) Diagram to summarize the mechanisms that Mehozumab‐DM1 inhibits hepatoma growth in HCC patients that are resistant to targeted therapy. Mehozumab‐DM1 was effectively internalized by cancer cells to release the payload, thereby inhibiting microtubule assembly and cell mitosis. Besides, Mehozumab‐DM1 induced RIPK‐dependent cell necroptosis by inhibiting the IκB kinase/NF‐κB pathway caused by inhibiting CD147‐induced changes in programmed cell death signaling. Furthermore, cell necroptosis causes cell membrane damage, the free payload was released outside the cell, thereby enhancing the bystander effect of ADC.

To further verify the biological effect of ADC on hepatoma cells, we performed Annexin V/7‐AAD apoptosis analysis on MHCC97‐H and Huh‐7 cell lines. Flow cytometry results (**Figure** **5**A) showed that treatment with 30 and 60 µg mL^−1^ ADC significantly increased the percentage of late apoptosis cells (necroptosis) in MHCC97‐H and Huh‐7 cell lines (*p <* 0.001 and *p <* 0.01, respectively). The necroptosis markers RIPK3 and MLKL were evaluated in Huh‐7 cells, revealing marked phosphorylation of RIPK3 and MLKL after 24 and 48 h of Mehozumab‐DM1 treatment (Figure 5B). We assumed that the activation of programmed cell death signaling was attributed to CD147‐induced changes in the apoptosis signaling pathway. Previous studies revealed that CD147 promotes the NF‐κB pathway to resist apoptosis.^[^ ^24^ ^]^ Therefore, we analyzed the differential genes of the NF‐κB pathway between the control and ADC‐treated cynomolgus monkeys. GSEA enrichment analysis (Figure 5C) suggested that ADC treatment caused a significant change in the NF‐κB pathway. This was supported by the immunoblotting results (Figure 5D), which confirmed decreased expression of phosphorylated inhibitor of κB kinase (phospho‐IKK) and phosphorylated nuclear factor κB p65 (phospho‐P65) after 24 and 48 h of Mehozumab‐DM1 treatment, suggesting NF‐κB pathway inhibition. It was reported that NF‐κB dynamics determine cell necroptosis through tumor necrosis factor alpha‐induced protein 3 (also known as A20).^[^ ^25^, ^26^ ^]^ Our results consistently showed decreased A20 levels following Mehozumab‐DM1 treatment (Figure 5D).

In summary, our investigations revealed that Mehozumab‐DM1 potently inhibits hepatoma growth in HCC patients. Mehozumab‐DM1 was effectively internalized by cancer cells to release the payload, thereby inhibiting microtubule assembly and cell mitosis. Besides, Mehozumab‐DM1 induced RIPK‐dependent cell necroptosis by inhibiting the IκB kinase/NF‐κB pathway caused by inhibiting CD147‐induced changes in programmed cell death signaling. Furthermore, because cell necroptosis causes cell membrane damage, the free payload was released outside the cell, thereby enhancing the bystander effect of ADC (Figure 5E).

## 3 Discussion

HCC is a highly therapy‐resistant and difficult‐to‐treat cancer. The majority of patients ultimately develop advanced‐stage HCC, at which point they may benefit from targeted therapy with sorafenib and lenvatinib as first‐line treatments and regorafenib, cabozantinib, and ramucirumab as second‐line treatments. However, the median overall survival of these patients is ≈1 year with the use of kinase inhibitors.^[^ ^27^ ^]^ Thus, there is an urgent need to identify predictors of the response to targeted therapy and to develop effective treatment strategies for patients with refractory HCC. ADCs are among the fastest‐growing oncology drug classes and have launched a new era of cancer treatment with remarkable achievements. Over the past decade, 15 ADC medications have been successfully brought to market, and more than 100 ADC medications are in different stages of clinical trials.^[^ ^28^, ^29^ ^]^ However, the targets of ADC medications are currently limited, and include HER2, CD22, and CD20, generally used to treat breast, lung, and gastric cancer. Novel targets for solid tumors, particularly liver cancer, are yet to be discovered, and the molecular mechanisms of therapeutic drugs still require investigation.^[^ ^30^, ^31^ ^]^ We previously demonstrated high levels of expression of the transmembrane protein CD147 in human HCC tissue, with a rate of positivity > 80%, which was significantly higher than in paracancerous tissue.^[^ ^32^ ^]^ In this study, we found that CD147 gene expression was markedly increased in early‐stage HCC, suggesting CD147 as a potential biomarker for the early diagnosis of liver cancer. More importantly, we observed higher levels of CD147 expression in patients who underwent PDL‐1/CTLA4 treatment and in those who did not respond to trastuzumab, bevacizumab, and sorafenib, implying an association between CD147 and patients’ primary resistance to targeted therapy. Bevacizumab treatment was reported to cause ischemia and hypoxia in tumor tissues due to its antiangiogenic effect, which further enhanced glycolysis and increased lactate excretion by tumor cells, leading to bevacizumab resistance through the epigenetic regulatory effect of histone lactylation in colon cancer.^[^ ^33^ ^]^ Many studies have demonstrated the important role of CD147 in regulating lactate intake and excretion by interacting with monocarboxylate transporters on the surface of tumor cells.^[^ ^34^, ^35^ ^]^ Additionally, CD147 colocalizes with VEGFR2 on the cell surface and upregulates the expression of VEGF subtypes and VEGFR2 by regulating HIF‐2α.^[^ ^36^, ^37^ ^]^ Therefore, CD147 may be involved in the mechanism of resistance to antiangiogenic therapy by inducing VEGF secretion, upregulating VEGFR2 expression, and promoting glucose and lactate transport in tumor cells. Thus, a combination of targeted CD147 therapy and antiangiogenic therapy may improve the curative effect for patients with refractory HCC who do not respond to first‐ or second‐line targeted therapy.

A mAb to CD147, HAb18, was developed to immunize mice in our laboratory by using cell suspension extracted from fresh human HCC tissues.^[^ ^38^ ^]^ We further developed Licartin ([^131^I] labeled HAb18), a radionuclide‐labeled antibody‐targeted drug for HCC treatment, which showed safety and efficacy in clinical applications and was approved by the China Food and Drug Administration for radioimmunotherapy of unresectable advanced or recurrent HCC.^[^ ^11^, ^12^ ^]^ In this study, we used a humanized HAb18 antibody, Mehozumab, which was engineered by grafting complementarity‐determining regions into the corresponding regions of a fixed human framework scaffold in our laboratory.^[^ ^17^ ^]^ To a large extent, this humanized antibody reduces the immune response and the effect of human anti‐mouse antibody (HAMA).^[^ ^18^, ^19^ ^]^ Moreover, the conjugation of Mehozumab to the small molecular drug DM1 avoids the risk of exposure to radioactivity caused by isotope labeling, meaning that specialized protection, which is required for Licartin treatment, is unnecessary. Additionally, Mehozumab‐DM1 is administered via intravenous injection, while Licartin requires a femoral vein puncture. Thus, Mehozumab‐DM is more feasible for widespread clinical application. Furthermore, because the DAR of Mehozumab‐DM1 is 5.80, which is an appropriate proportion of small molecular compounds to antibodies, its toxicity is controllable despite the potent killing activity of DM1.

Compared with rodents, NHPs are recognized as ideal animal models for the preclinical assessment of antitumor drugs.^[^ ^39^ ^]^ Previous pharmacodynamic evaluations of antitumor drugs mostly used transplanted tumor or orthotopic tumor mouse models. However, there are substantial differences between mice and humans, such as size, life span, pathophysiological mechanisms, and drug metabolism.^[^ ^40^ ^]^ Due to the high cost and the longer span for reproduction, cynomolgus monkeys are conventionally used for the safety evaluation of drugs. In this study, we used the CRISPR/Cas9 system to induce genomic editing in a cynomolgus monkey liver cancer model, which can appropriately imitate the tumor microenvironment and pathological characteristics of the different tumor stages and responses in humans, including tumorigenesis and drug response, resistance, and toxicity. Additionally, the CRISPR/Cas9‐expressing adenoviruses were directly delivered to the livers of cynomolgus monkeys through the intrahepatic portal vein under ultrasound guidance to achieve in situ gene editing, which significantly increased the efficiency and specificity of inducible gene editing.^[^ ^14^ ^]^ Moreover, the test reagents and technology used in this study are the same as those used in clinical tests in hospitals, thereby ensuring data reliability. Therefore, the preclinical efficacy and safety data of Mehozumab‐DM1 obtained in this study are extremely close to those in clinical patients, providing a reliable reference for clinical trials. Furthermore, the CRISPR‐mediated liver cancer model in our study was induced by *PTEN* and *TP5*3 mutations, which are predictors of a poor response to targeted therapy and immunotherapy and also predictors of poor survival.^[^ ^20^, ^21^, ^22^, ^23^ ^]^ Thus, our cynomolgus monkey model simulated patients with refractory liver cancer, and the potent in vivo efficiency of Mehozumab‐DM1 suggests that it is a promising clinical treatment for refractory HCC.

Mehozumab‐DM1 exhibits high inhibitory efficacy both in vitro and in vivo. The IC_50_ value in the HCC‐LM3 cell line was 3.597 × 10^−8^ m, and the overall response rate in the cynomolgus monkey liver cancer model was 100%. Mehozumab‐DM1 treatment at a low dosage (0.2 mg kg^−1^) resulted in a significant reduction in tumor volume. We believe that this potent inhibitory effect is attributable to three mechanisms. First, Mehozumab‐DM1 was rapidly and effectively internalized through receptor‐mediated endocytosis in HCC cell lines after a 1 h incubation. Upon internalization, the DM‐1 payload was released from the endosome and inhibited microtubule assembly and cell mitosis. Second, Mehozumab‐DM1 induced RIPK‐dependent cell necroptosis in tumor tissue from the cynomolgus monkey and in HCC cell lines. We speculate that cell necroptosis was caused by antibody blocking of CD147‐induced changes in the programmed cell death signaling pathway. We further confirmed that Mehozumab‐DM1 induced RIPK‐dependent necroptosis via IκB kinase/NF‐κB pathway inhibition. Third, cell necroptosis leads to an enhanced bystander effect. A typical marker of cell necroptosis is the phosphorylation of MLKL on the cell surface,^[^ ^41^ ^]^ which destabilizes and ultimately breaks down the cell membrane, releasing the free payload outside the cell, thereby enhancing the bystander effect. We also performed a preliminary safety evaluation of Mehozumab‐DM1 in cynomolgus monkey. There was no obvious body weight loss, and the routine blood tests did not reveal any drug‐induced liver injury, anemia, or infection caused by the ADC medication. These results suggest that patients with HCC may have a good tolerance to Mehozumab‐DM1 when treated with the correct dosage.

An interesting finding of this study was that Mehozumab‐DM1 treatment‐induced cell necroptosis in cynomolgus monkey liver cancer model. Necroptosis is a caspase‐independent necrotic cell death pathway that is regulated by receptor‐interacting protein (RIP) 1 kinase and its downstream mediator RIP3 kinase. It is characterized by the early loss of plasma membrane integrity, leakage of intracellular contents, and swollen organelles.^[^ ^41^, ^42^ ^]^ Accumulating evidence has demonstrated that CD147 promotes tumor progression by inhibiting cancer cell apoptosis.^[^ ^43^ ^]^ CD147 interacts with CD44, activating the JAK/STAT3 signaling pathway, which inhibits apoptosis.^[^ ^44^ ^]^ CD147 also inhibits apoptosis by forming a tetrameric complex with Xkr8, inhibiting its function and blocking PtdSer exposure during apoptosis.^[^ ^45^ ^]^ CD147 regulates IGFBP2 expression, leading to apoptosis inhibition by regulating caspase‐3 activation and the PTEN/PI3K/Akt pathway.^[^ ^46^ ^]^ A recent study reported that treatment with the anti‐CD147 mAb M6‐1D4 might lead to oncosis‐induced necroptosis in HCC cell lines.^[^ ^47^ ^]^ In our study, we observed typical cell necroptosis with mitochondrial swelling and endoplasmic reticulum expansion by electron microscopy of thein cynomolgus monkey liver cancer model following Mehozumab‐DM1 treatment. Necroptosis was reported to be dynamically regulated by the NF‐κB/RelA signaling pathway.^[^ ^25^, ^48^ ^]^ Moreover, our study on rheumatoid arthritis indicated that CD147 downregulation both increased the apoptotic rate and inhibited IKK/IκB kinase/NF‐κB pathway‐dependent proinflammatory cytokine secretion.^[^ ^24^ ^]^ Therefore, we speculate that Mehozumab‐DM1 induces necroptosis by inhibiting the NF‐κB pathway. Our RNA‐seq analysis results consistently suggested the suppression of NF‐κB signaling after ADC treatment, which was supported by our immunoblotting results. This study discloses a novel mechanism of Mehozumab‐DM1‐induced cell necroptosis due to CD147 blocking causing changes in the programmed cell death signaling pathway, thereby enhancing the bystander effect of ADCs. Although further safety assessments of Mehozumab‐DM1 in animals and humans are necessary, this preclinical study provides strong evidence for the promotion of further clinical studies.

## 4 Conclusion

This study successfully developed a humanized anti‐CD147 mAb conjugated to DM1 that inhibits hepatoma growth in vitro and in vivo. Mehozumab‐DM1 is effectively internalized by cancer cells to release the DM1 payload, which inhibits microtubule assembly and cell mitosis. Mehozumab‐DM1 also induces RIPK‐dependent cell necroptosis through inhibiting the IκB kinase/NF‐κB pathway caused by the inhibition of CD147‐induced changes in programmed cell death signaling. Furthermore, cell necroptosis damages the cell membrane, the free payload is released outside the cell, thereby enhancing the bystander effect. Thus, Mehozumab‐DM1 is a potential optimal therapeutic for HCC patients who are resistant to targeted therapy.

## 5 Experimental Section

### Study Design

Experiments were designed to evaluate anti‐CD147 ADC Mehozumab‐DM1 as a promising treatment for refractory HCC. The internalization efficacy and in vitro activity of Mehozumab‐DM1 in HCC cell lines were investigated, using naked Mehozumab as a control. To confirm activation of the necroptosis signaling pathway in HCC cell lines treated with the ADC, immunoblotting was used. A cynomolgus monkey liver cancer model was used to evaluate the in vivo efficacy of Mehozumab‐DM1 and perform a preliminary safety assessment. Qualified cynomolgus monkey liver cancer models were selected and randomly assigned to one of three treatment groups: 1) ADC intravenous injection 0.2 mg kg^−1^ group (*n* = 3), 2) ADC intravenous injection 1.0 mg kg^−1^ group (*n* = 3), or 3) physiological saline control group (*n* = 2). During the 8‐week ADC treatment, the tumor volume and body weight were recorded, and analyzed the serum AFP level and the results of liver function and routine blood tests in the model monkeys. To further investigate the antitumor mechanism of Mehozumab‐DM1, Electron microscopy was used to assess the tumor tissues from the model monkeys, and performed RNA‐seq analysis was also performed on the tumor tissues. All animal experiments were performed in accordance with the People's Republic of China Legislation Regarding the Use and Care of Laboratory Animals. All study protocols were approved (approval number: 202101030) by the Animal Welfare and Ethics Committee of Guangxi Medical University (Nanning, Guangxi Province, China). The researchers were not blinded during the experiments.

### Generation of an In Situ CRISPR‐Mediated Cynomolgus Monkey Liver Cancer Model

A CRISPR‐mediated cynomolgus monkey liver cancer model was previously established.^[^ ^14^ ^]^ Briefly, *PTEN* and *TP5*3 genes were selected as two target genes to produce mutations for the construction of cynomolgus monkey models of primary and metastatic liver cancer. ultrasound guidance was used to inject the CRISPR/Cas9 adenoviruses into the cynomolgus monkey liver through the intrahepatic portal vein, leading to the rapid generation of NHP models of liver cancer.

### Cell Lines

Human HCC cell lines HCC‐LM3, MHCC97‐H, and Huh‐7 were purchased from the Institute of Biochemistry and Cell Biology, Chinese Academy of Sciences. All cell lines underwent short tandem repeat DNA profiling and were authenticated before use in the experiments. They were cultured in RPMI 1640 medium supplemented with 10% fetal bovine serum (FBS) at 37 °C under 5% CO_2_.

### Conjugation of Mehozumab‐DM1

The Mehozumab antibody was provided by Jiangsu Pacific Meinuoke Bio‐Pharmaceutical Co., Ltd. After dissolving SMCC−DM1 in dimethylacetamide to a concentration of 10 mg mL^−1^, it was slowly added to the Mehozumab antibody and mixed well. Then the mixture was placed on a thermostatic shaker (130 rpm) at 25 °C for 12 h in the dark and allowed to react. Next, the ADC conjugate was concentrated and purified using a HiLoad 16/600 Superdex 200 pg chromatographic column (Cytiva) on an āKTA Purifier 100 chromatography system. The Mehozumab‐DM1 purity was analyzed using HPLC, and the drug‐to‐antibody ratio (DAR) of the Mehozumab‐DM1 was determined using UV spectrophotometric analysis and quadrupole time‐of‐flight LC/MS analysis.

### Imaging Flow Cytometry

Cells (1 × 10^6^) were seeded in each well of six‐well chamber slides and allowed to attach overnight. Then IgG, Mehozumab, and Mehozumab‐DM1 were labeled with FITC (cat#: 46 425; Thermo Fisher). Cells were incubated with 20 µg labeled IgG, Mehozumab, and Mehozumab‐DM1 in 1% FBS medium at 37 °C for 1 h. Finally, the cells were collected, washed twice with precooled phosphate‐buffered saline (PBS) (1000 rpm, 5 min), and resuspended in 300 µL PBS. The rate of antibody internalization by the treated cells was measured using an Amnis ImageStreamX Mark II imaging flow cytometer (Luminex, Austin, TX, USA).

### Immunohistochemical Staining

Tumor tissues of cynomolgus monkey liver cancer model were paraffin‐embedded. To prepare for immunohistochemical staining, paraffin sections were dewaxed, followed by antigen retrieval with 10 mmol L^−1^ citrate buffer (pH 6.0). Deparaffinized sections were treated with methanol containing 3% hydrogen peroxide for 15 min. After washing with PBS, the sections were incubated with blocking serum for 30 min. Then, the sections were incubated with Mehozumab‐DM1 (dilution 1:3000) at 4 °C overnight. Finally, we used a General SP kit (cat#: SP9000; ZSGB‐BIO) for immunoperoxidase staining followed by treatment with 3,3′‐diaminobenzidine (cat#: ZLI‐9019; ZSGB‐BIO) to detect the target proteins. Nuclei were counterstained using hematoxylin.

### Cell Counting Kit‐8 Assay

Human HCC cell lines HCC‐LM3, MHCC97‐H, and Huh‐7 were seeded in 96‐well plates (3000 cells per well). After the cells had adhered to the walls, different concentration gradients of Mehozumab‐DM1 diluent, Mehozumab diluent, and IgG were added, and the cells were cultured in a 37 °C incubator under 5% CO_2_ for 72 h. ThenCCK8 (cat#: C005; Topscience Biology) was used to determine cell viability. Per the manufacturer's instructions, 10 µL of CCK8 reagent was added to each well followed by incubation for 1 h. The absorbance was measured at 450 nm, and used to calculate the corresponding IC_50_ value using GraphPad Prism v8.0 software (GraphPad Software Inc., San Diego, CA).

### In vivo Inhibitory Efficacy Evaluation

The in vivo therapeutic efficacy of Mehozumab‐DM1 was tested in our CRISPR‐mediated cynomolgus monkey liver cancer model. Qualified cynomolgus monkey liver cancer models were selected and randomly divided into three groups (ADC intravenous injection 0.2 mg kg^−1^, ADC intravenous injection 1.0 mg kg^−1^, or physiological saline control). The tumor volume was determined using an ultrasound scan. When the tumor diameter reached ≈1.00 cm, the treatment was administered weekly for 8 weeks according to the assigned group. The tumor volume was calculated as V (cm^3^) = 0.5 × L × W^2^, where V is the tumor volume, L is the tumor length, and W is the tumor weight. The calculated tumor volumes were then used to determine the tumor volume inhibition rate as follows: tumor volume inhibition rate = (tumor volume after treatment – tumor volume before treatment)/tumor volume before treatment × 100%. The body weight of the monkeys in all groups was recorded weekly for 10 weeks. Blood samples (3 mL peripheral blood) were collected from the monkeys before and at 4 and 8 weeks after the ADC treatment and analyzed to determine the serum levels of AFP, AST, and ALT using a clinical testing kit at the First Affiliated Hospital of Guangxi Medical University.

### Electron Microscopy

The tissue samples were cut into 1 mm^3^ pieces and fixed in PLP Fixing Solution (cat#: G2220, Solarbio) for at least 24 h. After washing with PBS, the sections were treated with osmic acid for 1.5 h. Then they were dehydrated through an ascending alcohol gradient and soaked in acetone for 15 min. After embedding and polymerization with epoxy resin overnight, sections were cut using an ultramicrotome and fixed to a perforated copper grid. Finally, each section was stained with lead citrate and uranium solution for 10 min, washed with water, and dried. The ultrastructure was observed using a JEM 1230 transmission electron microscope (JEOL Ltd.).

### RNA Sequencing

Total RNA was extracted from the tumor tissue of monkeys in the physiological saline control group and monkeys in the 1.0 mg kg^−1^ ADC treatment group. It was sent to Gene Denovo Biotechnology (Guangzhou, China) for RNA‐seq and subsequent data analysis, including GO analysis and GSEA, using the OmicsShare platform.

### Flow Cytometry Analysis

MHCC97‐H and Huh‐7 human HCC cell lines were seeded in six‐well plates (1–2 × 10^6^ cells per well). After the cells had adhered to the well walls, different concentration gradients of Mehozumab‐DM1 diluent, Mehozumab diluent, and IgG were added, and the cells were cultured in a 37 °C incubator under 5% CO_2_ for 72 h. Apoptosis was measured using a PE Annexin V Apoptosis Detection Kit with 7‐AAD (cat#: 640934; BioLegend), according to the manufacturer's instructions. Briefly, the cells were labeled with Annexin V‐PE and 7‐AAD for 20 min at room‐temperature in the dark. Then they were evaluated using an LSRFortessa flow cytometer (BD Biosciences), and the acquired data were analyzed with FlowJo v10 software.

### Immunoblotting

Cells (5 × 10^5^) were seeded in each well of six‐well chamber slides and allowed to attach overnight. Then cells were incubated with 50 µg/mL ADC at 37 °C in 10% FBS medium for 0, 24, and 48 h, or with 50 µg mL^−1^ IgG‐DM1 and Mehozumab at 37 °C in 10% FBS medium for 48h. Following collection, the cells were lysed on ice for 15 min with 100 µL RIPA lysis buffer (cat#: P0013B; Beyotime) containing 1% PMSF (cat#: ST505; Beyotime) and then centrifuged (16000 × *g*, 4 °C, 20 min). The protein supernatant was obtained, quantified with BCA(cat#: P0009; Beyotime), then added with 5 × SDS Loading Buffer and cooked at 100 °C for 8 min. The protein samples were separated by SDS‐PAGE and transferred to PVDF membranes (0.45 µm; cat# IPVH00010; Millipore). After blocking the membranes with 5% skim milk powder for 1 h at room‐temperature, they were incubated overnight at 4 °C with the following primary antibodies: RIP3 (1:1000; cat#: ab56164; Abcam), phospho‐RIP3 (1:800; cat#: 93654; Cell Signaling Technology), MLKL (1:1000; cat#: ab184718; Abcam), phospho‐MLKL (1:800; cat#: 91689; Cell Signaling Technology), IKK (1:800; cat#: ab178870; Abcam), phospho‐IKK (1:800; cat#: 2697; Cell Signaling Technology), p65 (1:1000; cat#: 10745‐1‐AP; Proteintech), phospho‐p65 (1:1000; cat#: 3033; Cell Signaling Technology,), A20 (1:1000; cat#: 5630; Cell Signaling Technology), and mouse GAPDH monoclonal antibody (1:4000; cat#: 60004‐1‐Ig; Proteintech). The membranes were washed at least three times with TBST and incubated with the appropriate horseradish peroxidase‐conjugated secondary antibody (1:5000; cat#: 31430; Invitrogen or cat#: 31460, Invitrogen) for 1 h at room‐temperature. Images were acquired using a ChemiDoc imaging system (Bio‐Rad Laboratories).

### Statistical Analysis

All statistical analyses were performed using GraphPad Prism software, version 8.0 (San Diego, CA). The sample size (n) for each analysis is shown in the appropriate figure. Student *t*‐test was used to compare the mean expression levels between two groups, and One‐way ANOVA was used to compare the mean between three or more subgroups. Kaplan–Meier curves and log‐rank analyses were used in the survival analysis. One‐way ANOVA was used to compare differences in the tumor volume inhibition rate between the model monkeys in the control group and those in the treatment groups. A post‐hoc test was used to verify differences in the tumor volume inhibition rate between the control group and each treatment group of the cynomolgus monkey liver cancer model. Two‐sided p values < 0.05 were considered statistically significant.

## Conflict of Interest

The authors declare no conflict of interest

## Supporting information



Supporting Information

## Data Availability

The data that support the findings of this study are available on request from the corresponding author. The data are not publicly available due to privacy or ethical restrictions.
