# Supplementary material for: An Anti‐CD147 Antibody−Drug Conjugate Mehozumab‐DM1 is Efficacious Against Hepatocellular Carcinoma in Cynomolgus Monkey
Source: Adv Sci (Weinh). 2025 Feb 22;12(15):2410438. doi: 10.1002/advs.202410438 (PMC12005782; doi:10.1002/advs.202410438)
Supplement: Supplementary file 1 — Supporting Information [file ADVS-12-2410438-s001.pdf]

ADVANCED SCIENCE
Open Access

Supporting Information

for *Adv. Sci.*, DOI 10.1002/advs.202410438

An Anti-CD147 Antibody–Drug Conjugate Mehozumab-DM1 is Efficacious Against Hepatocellular Carcinoma in Cynomolgus Monkey

Wan Huang, Liping Zhong, Ying Shi, Qingzhi Ma, Xiangmin Yang, Hongmei Zhang, Jing Zhang, Ling Wang, Kun Wang, Jingzhuo Li, Jie Zou, Xu Yang, Liu Yang, Qingmei Zeng, Lin Jing, Zhi-Nan Chen* and Yongxiang Zhao**

Supplementary Information

An Anti-CD147 Antibody−Drug Conjugate Mehozumab-DM1 is Efficacious Against Hepatocellular Carcinoma in Cynomolgus Monkey

Wan Huang[1,2*], Liping Zhong[3], Ying Shi[1,2], Qingzhi Ma[1,2], Xiangmin Yang[1,2], Hongmei Zhang[4], Jing Zhang[5], Ling Wang[6], Kun Wang[1,2], Jingzhuo Li[1,2], Jie Zou[1,2], Xu Yang[1,2], Liu Yang[1,2], Qingmei Zeng[1,2], Lin Jing[1,2], Zhi-Nan Chen[1,2*], Yongxiang Zhao[3*]

[1]Department of Cell Biology, National Translational Science Center for Molecular Medicine, Fourth Military Medical University, Xi'an, Shaanxi, China

[2]State Key Laboratory of New Targets Discovery and Drug Development for Major Diseases, Xi'an, Shaanxi, China

[3]State Key Laboratory of Targeting Oncology, National Center for International Research of Biotargeting Theranostics, Guangxi Key Laboratory of Biotargeting Theranostics, Collaborative Innovation Center for Targeting Tumor Diagnosis and Therapy, Guangxi Medical University, Nanning, Guangxi, China

[4]Department of Clinical Oncology, Xijing Hospital, Fourth Military Medical University, Xi'an, Shaanxi, China

[5]Department of Pathology, Xijing Hospital, The Fourth Military Medical University, Xi'an, Shaanxi, China.

[6]Department of Health Statistics, School of Preventive Medicine, Fourth Military Medical University, Xi'an, Shaanxi, China.

These authors contributed equally: Wan Huang, Liping Zhong, Ying Shi, Qingzhi Ma, Xiangmin Yang

***CORRESPONDING AUTHORS**

Yongxiang Zhao, zhaoyongxiang@gxmu.edu.cn.

Zhi-nan Chen, znchen@fmmu.edu.cn.

Wan Huang, huangwan@fmmu.edu.cn.

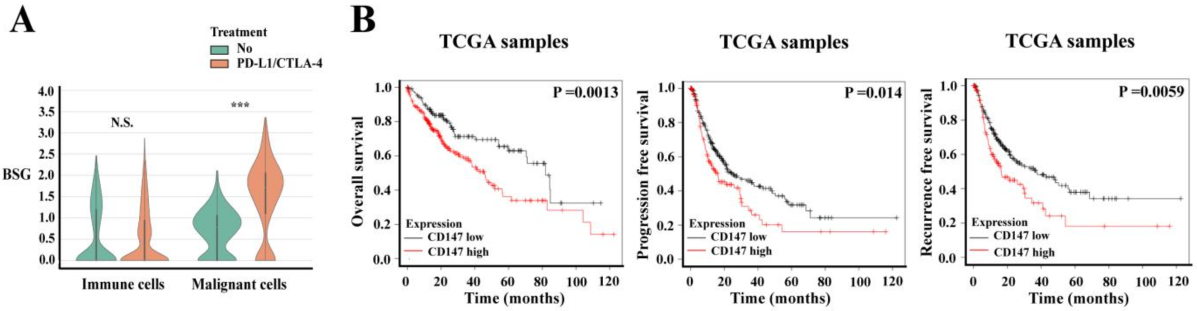

Figure S1 Expression of CD147 was significantly correlated with poor survival of HCC patients. A Gene expression of CD147 (BSG) was quantitatively compared in the tumor tissues from patients received PDL-1/CTLA4 treatment and patients who have not received immunotherapy based on TISCH2 scRNA-seq database. **B** Kaplan Meier curves and log rank analyses to investigate the correlation of CD147 expression with overall survival, progression free survival and recurrence free survival based on Kaplan Meier Plotter dataset.

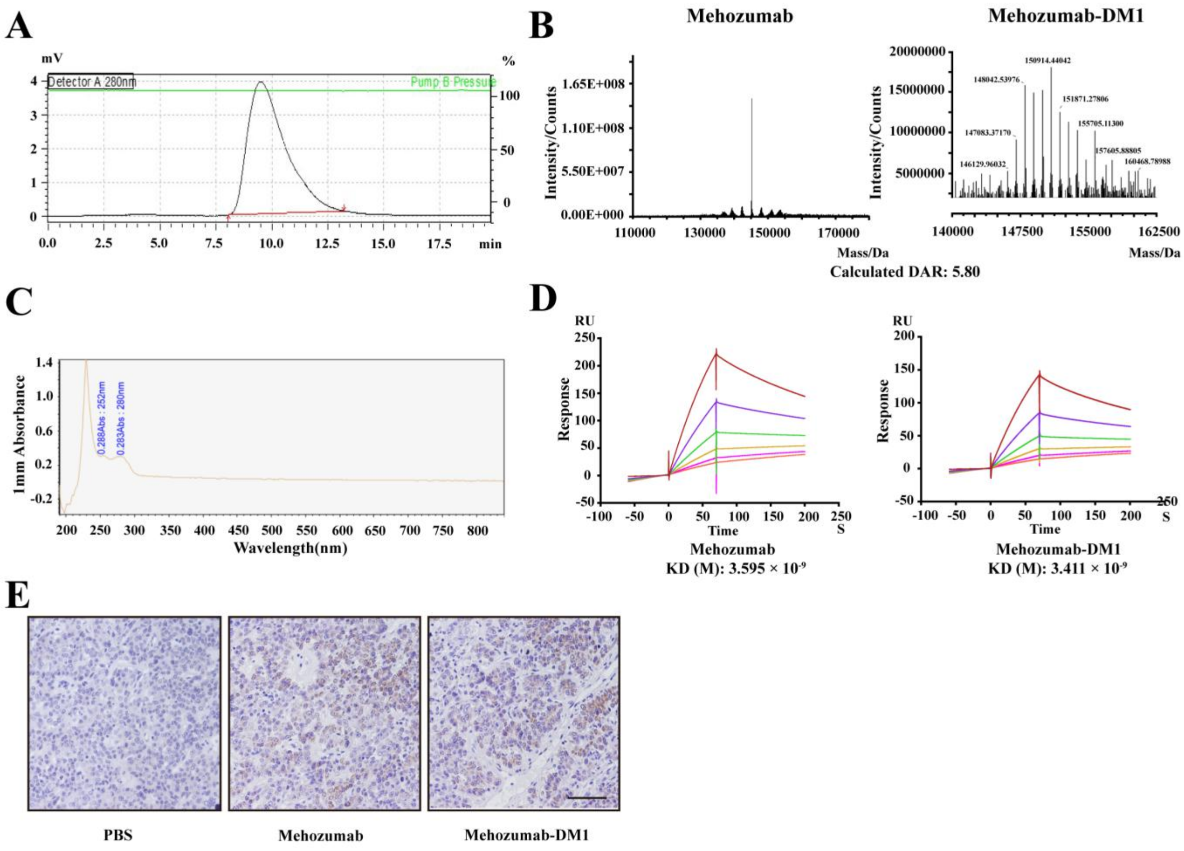

Figure S2 Preparation and characterization of Mehozumab-DM1. A High performance liquid chromatography analysis showed that the purity of the Mehozumab-DM1 was > 99.99%. **B** Quadrupole time-of-flight LC/MS analysis to detect the DAR of the Mehozumab-DM1. The mass spectrogram for unconjugated Mehozumab (left) and Mehozumab-DM1 (right) were shown. The calculated DAR was 5.80. **C** Typical absorbance spectrum by UV spectrophotometric analysis to detect the drug-to-antibody ratio (DAR) of the Mehozumab-DM1. **D** Surface Plasmon Resonance (SPR) analysis to detect the affinity of Mehozumab-DM1 binding to CD147 compared to parent antibody Mehozumab. **E** Immunohistochemical staining to detect the affinity and specificity of Mehozumab-DM1 binding to CD147 in the tumor tissue of cynomolgus monkey compared to naked Mehozumab antibody. Scale bars represent 50 μm.

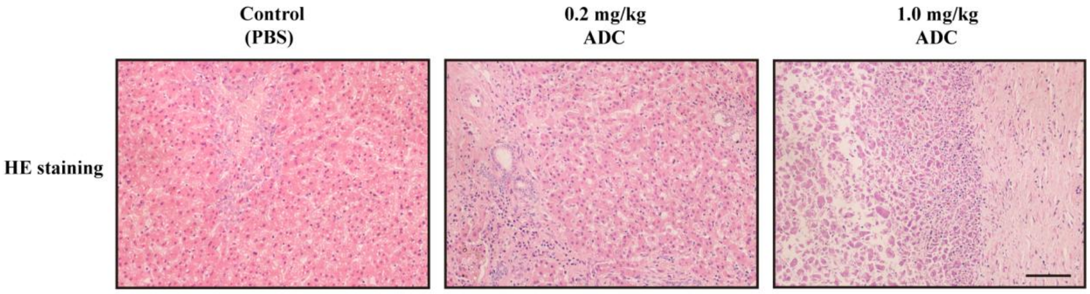

Figure S3 H&E staining of tumor tissues from control and ADC treatment cynomolgus monkey. A distinct liquefactive necrosis was observed in the tumor tissue of 0.2 mg/kg and 1 mg/kg ADC treatment cynomolgus monkey. Scale bars represent 50 μm.

Table S1 Tumor size of each cynomolgus monkey before and after Mehozumab-DM1 treatment

Dose group	Monkey No.	Before treatment			After treatment		
		Long diameter	Short diameter	Volume	Long diameter	Short diameter	Volume
**Physiological saline**	1	1.26	0.89	0.50	1.49	1.47	1.61
**Physiological saline**	2	1.08	0.66	0.24	1.30	0.83	0.45
**0.2 mg/kg**	3	1.45	1.34	1.30	0.69	0.38	0.05
**0.2 mg/kg**	4	1.35	1.19	0.96	0.96	0.77	0.28
**0.2 mg/kg**	5	1.71	1.17	1.17	0.56	0.42	0.05
**1 mg/kg**	6	0.78	0.72	0.20	0.00	0.00	0.00
**1 mg/kg**	7	3.16	1.60	4.04	1.45	1.22	1.08
**1 mg/kg**	8	0.81	0.54	0.12	0.00	0.00	0.00

Table S2 Blood routine test results of each cynomolgus monkey

Dose group	Monkey No.	RBC ($\times10^{12}$/L)			WBC ($\times10^{9}$/L)			PLT ($\times10^{12}$/L)		
		Before treatment	Treatment for 4 weeks	Treatment for 8 weeks	Before treatment	Treatment for 4 weeks	Treatment for 8 weeks	Before treatment	Treatment for 4 weeks	Treatment for 8 weeks
**physiological saline**	1	6.42	6.96	7.89	9.92	11.44	12.85	261	273	253
**physiological saline**	2	12.16	13.39	11.97	8.61	9.69	11.69	298	316	298
**0.2 mg/kg**	3	11.36	9.67	10.15	7.59	8.44	9.11	245	286	195
**0.2 mg/kg**	4	7.83	8.47	8.03	10.38	8.59	8.98	327	304	261
**0.2 mg/kg**	5	8.5	9.01	8.26	6.59	7.08	8.13	312	261	219
**1 mg/kg**	6	10.28	11.81	9.31	11.83	9.76	9.04	287	291	185
**1 mg/kg**	7	8.15	9.52	10.14	12.32	16.42	11.93	258	226	143
**1 mg/kg**	8	10.18	11.01	11.61	9.52	10.16	7.16	304	255	193

Dose group	Monkey No.	NE%			LY%		
		Before treatment	Treatment for 4 weeks	Treatment for 8 weeks	Before treatment	Treatment for 4 weeks	Treatment for 8 weeks
**physiological saline**	1	55.7	66.1	69.1	27.8	23.1	23.1
**physiological saline**	2	63.2	70.2	73.2	23.7	26.5	26.5
**0.2 mg/kg**	3	61.9	65.8	72.5	27.5	34.9	34.9
**0.2 mg/kg**	4	49.5	56.7	69.4	26.9	32.3	32.3
**0.2 mg/kg**	5	51.9	68.3	60.8	18.4	28.7	28.7
**1 mg/kg**	6	63.1	73.1	58.2	33.6	40.4	40.4
**1 mg/kg**	7	48.8	69.2	71.7	29.1	35.6	35.6
**1 mg/kg**	8	56.5	69.9	73.5	29.2	35.5	35.5

RBC, red blood cell; WBC, white blood cell; PLT, platelet; EN%, percentage of eosinophils; LY%, percentage of lymphocyt
